# Ethnic disparities in prevalence and clustering of cardiovascular disease risk factors in rural Southwest China

**DOI:** 10.1186/s12872-019-1185-1

**Published:** 2019-08-19

**Authors:** Li Hui-Fang, Le Cai, Xu-Ming Wang, Allison Rabkin Golden

**Affiliations:** 1 Cheng Gong New City, School of Public Health, Kunming Medical University, 1168 Yu Hua Street Chun Rong Road, Kunming, 650500 China; 2 The First Affiliated Hospital of Kunming Medical University, Kunming, China

**Keywords:** Cardiovascular disease, Clustering of CVD risk factors, Ethnicity, Socioeconomic status, China

## Abstract

**Background:**

This study examines how prevalence and clustering of cardiovascular disease (CVD) risk factors differ by ethnicity and socioeconomic status (SES) among rural southwest Chinese adults.

**Methods:**

A cross-sectional survey of 7027 adults aged ≥35 years of Han and four ethnic minority group descent (Na Xi, Li Shu, Dai, and Jing Po) was used to derive prevalence of tobacco smoking and exposure to secondhand smoke (SHS) as well as alcohol consumption and physical activity data. Anthropometric measurements were also taken, including height, weight, and waist and hip circumference, as well as blood pressure (BP) and fasting blood glucose (FBG) measurements.

**Results:**

Current smoking and drinking status were the top two CVD risk factors in the study population. Dai ethnic minority participants had the highest prevalence of hypertension, obesity, and central obesity, whereas Jing Po ethnic minority participants had the highest prevalence of current smoking status, SHS exposure, and current drinking status (*P* < 0.01). Han participants had the highest prevalence of diabetes and physical inactivity (*P* < 0.01). 11.1% of all participants did not have any of the studied CVD risk factors, while 68.6% of Han, 60.2% of Na Xi, 50.7% of Li Shu, 82.2% of Dai, and 73.0% of Jing Po participants had clustering of two or more CVD risk factors. Prevalence of CVD risk factor clusters increased with age (*P* < 0.01). Males and individuals with lower education levels and lower annual household income were more likely to have CVD risk factors than their counterparts (*P* < 0.01).

**Conclusion:**

Clustering of CVD risk factors is common in rural southwest China. Ethnicity and individual SES significantly impact prevalence of CVD risk factors and their clustering.

## Background

Cardiovascular disease (CVD) is the leading cause of death worldwide, in both developed and developing countries [1]. Nearly 30% of all deaths globally (approximately 17.5 million people) resulted from CVD in 2008, and the number of fatalities is expected to increase to 23.3 million by 2030 [2]. In China, rapid economic growth, an aging population, and lifestyle changes in the last two decades have caused both the morbidity and mortality of CVD to rise rapidly [3]. This rise is projected to persist, with an estimated annual increase of 21.3 million CVD events and 7.7 million CVD-related deaths by 2030 [4]. This growing pandemic of CVD poses a major challenge for the Chinese healthcare system.

The majority of CVD risk factors are related to lifestyle and therefore potentially modifiable and controllable [5, 6]. As in other countries, hypertension, diabetes, being overweight or obese, smoking, alcohol consumption, and physical inactivity are well recognized established major risk factors for CVD in China [7, 8]. Furthermore, previous Chinese studies indicate that CVD risk factor clusters are common in China and that the prevalence of CVD risk factors has increased rapidly [9, 10], translating into a future increase in CVD burden in China [11].

China is a multiethnic nation, with 56 distinct ethnicities. The Han ethnicity accounts for the majority, 92%, of the population. The other 55 ethnicities are considered national minorities. Numerous studies have explored racial and ethnic disparities in prevalence and clustering of CVD risk factors worldwide [12, 13], revealing CVD risk factors differ significantly between ethnic groups. However, limited investigations have been conducted to examine ethnic differences in prevalence and clustering of CVD risk factors in China [14, 15]. As many ethnic minority groups in China are socioeconomically disadvantaged relative to the Han population, an improved understanding of ethnic disparities in central risk factors that contribute to CVD is critical to effectively implementing appropriate CVD intervention strategies in China.

Yunnan Province is located in southwest China and is both a production and consumption hub for tobacco products. It is also home to the most ethnic minority groups in China, with residents of 25 different ethnic minorities across the province, 15 of which live only in Yunnan. Culture, lifestyle habits, and genetic background differ among these unique ethnic minorities. Notable for the present study, alcohol consumption, cigarette smoking, and secondhand smoke (SHS) exposure are comparatively prevalent in these populations [16]. However, data on the prevalence and clustering of CVD risk factors in these ethnic minority populations is limited. Thus, this study aimed to examine ethnic disparities in prevalence and clustering of eight major CVD risk factors— hypertension, diabetes, cigarette smoking, SHS exposure, alcohol consumption, obesity, central obesity, and physical inactivity— among the Han majority and four ethnic minority groups (Dai, Li Shu, Jing Po, and Na Xi) in rural southwest China.

## Methods

### Study area and population

The present study employed a community-based cross-sectional in-person interview and examination. Research was conducted between 2015 and 2017 in one majority Han-populated county and four majority ethnic minority-populated areas of Yunnan Province. Residents were selected for participation in the study through four-stage stratified random sampling. This sampling method has previously been described in detail [17].

### Pilot study and modification of questionnaire

Pre-tests of the study questionnaire were conducted with 30 individual, in-person interviews of residents aged ≥35 years in a rural region of Yunnan Province. The results of this test were used to revise the questionnaire, specifically, to modify questions discovered to be ambiguous or inconsistent.

### Data collection and measurement

Individual, in-person interviews by trained interviewers with a structured questionnaire were conducted for each consenting participant. Demographic information, including age, sex, ethnicity (Han, Dai, Li Shu, Jing Po, and Na Xi), annual income (US dollars), level of education (years), smoking and drinking habits, and physical activity was taken for each participant by self-report. All participants’ anthropometric measurements, namely, height, weight, and waist and hip circumference, as well as blood pressure (BP) and fasting blood glucose (FBG) were also recorded.

For BP measurements, three consecutive measurements were made, in line with the procedure recommended by the American Heart Association [18]. Recorded BP levels for the present study were derived from the average of these three BP readings. This procedure has previously been detailed [17].

Standardized procedures, as outlined in the World Health Organization (WHO) STEPS manual, were used to measure height, weight, and waist and hip circumference [19]. Body mass index (BMI) was calculated as weight in kilograms divided by height in meters squared.

Healthcare workers working at existing local community clinics performed all blood sample collection. The fasting blood glucose measurement method used has been previously detailed [20].

### Definitions

Hypertension was defined as a mean systolic blood pressure (SBP) ≥140 mmHg, diastolic blood pressure (DBP) ≥90 mmHg, and/or use of antihypertensive medications [21]. A previous diagnosis of hypertension by a health professional was also considered grounds for establishing hypertension status in this study. Diabetes mellitus was defined as a FBG ≥7.0 mmol/l (126 mg/dl), reported use of antidiabetic medications, or reported previous diagnosis of diabetes by a health professional [22].

Obesity was defined as a BMI of 28 kg/m^2^ or greater, while central obesity was defined as a waist circumference (WC) of more than 90 cm in men and more than 80 cm in women, based on WHO recommendations for Asian adults [23].

Participants who had smoked at least 100 cigarettes in their lifetime and participants who smoked any form of tobacco product on a daily basis during the survey period were defined as current smokers. Exposure to SHS was defined as non-smokers who reported being exposed to another person’s tobacco smoke at home or at work for a minimum of 15 min at least 1 day per week. Participants who drank alcohol regularly on 12 or more days in the 12 months preceding the survey were defined as current drinkers.

Physical activity was assessed in two ways: by frequency (number of times a participant exercised in the week prior to the survey) and self-reported intensity of exercise per day. Physical inactivity was defined as engaging in moderate intensity exercise (such as running, cycling, walking, and jogging) less than 5 days a week for less than or equal to 30 min per day, in accordance with WHO recommendations for physical activity in adults [24].

Clustering of CVD risk factors was evaluated by determining the presence of the eight major factors under investigation in the present study (i.e., presence of 0, 1, 2 or ≥ 3 risk factors) [25].

### Statistical analysis

Data were double entered into an EpiData Entry 3.1 electronic database and analyzed with SPSS 22.0 software. Annual household income was divided into two categories: low (<$800 US) and high (≥$800 US), with the median value as the cutoff point. Level of education was also classified into two categories: primary (grade 1–6) or lower and middle (grade 7–9) or higher. Mean values of height, weight, FBG, BP, BMI, and waist circumference were expressed as mean ± standard deviations (*x* ± *s*). Categorical variables were presented as counts and percentages. The 2010 China Population Census was used to calculate sampling weights and our sampling scheme. The prevalence rates of CVD risk factors and clustering of CVD risk factors, however, were estimated based on weighted proportions. A direct method using China’s adult population aged 35 years and over, using 2010 as the standard population, was used to calculate age-standardized prevalence rates of risk factors and was computed as a percentage with a 95% confidence interval (CI). Data were analyzed with descriptive analysis techniques, chi-squared test and one-way ANOVA. Chi-square tests for independence were conducted for each risk factor across sex, ethnicity group, and socioeconomic status, while one-way ANOVA were used to analyze continuous measures across the ethnic groups. All statistical significance decisions were based on two-tailed *P* values of < 0.05.

## Results

A total of 7500 individuals aged ≥35 years were invited to participate in the present study from name lists of eligible individuals obtained from township leaders from the 15 townships. Of these, 7027 consented to participate, a response rate of 93.7%.

Table 1 displays the demographic characteristics and mean values of blood pressure, fasting blood glucose, and anthropometric measurements of the study population. 3434 (48.9%) of participants were male and 3593 (51.1%) were female. 49.4, 50.6, 45.9, 49.2, and 49.1% of males were from Han, Dai, Na Xi, Jing Po, and Li Shu ethnicities, respectively. Highest annual household incomes were recorded in Han participants, who also had the highest level of education relative to the other four studied ethnicities (*P* < 0.01). Jing Po ethnic minority participants had the lowest mean height of the five studied ethnicities (*P* < 0.01), whereas the Li Shu ethnic minority participants had both the lowest mean weight and BMI (*P* < 0.01). Dai ethnic minority participants had the highest mean systolic and diastolic BP and FBG (*P* < 0.01).

**Table 1:** Demographic characteristics and mean value of BP, FBG, and anthropometric measurements among the study population

Characteristics	Han ethnic majority (*n* = 1495)	Na Xi ethnic minority (*n* = 1402)	Li Shu ethnic minority (*n* = 1366)	Dai ethnic minority (*n* = 1397)	Jing Po ethnic minority (*n* = 1367)	All (*n* = 7027)
Sex (n, %)						
Male	757 (50.6)	644 (45.9)	675 (49.4)	686 (49.1)	672 (49.2)	3434 (48.9)
Female	738 (49.4)	758 (54.1)	691 (50.6)	711 (50.9)	695 (50.8)	3593 (51.1)
Age (years, mean ± SD)	54.2 ± 12.2	53.9 ± 13.1	50.8 ± 12.0	54.9 ± 12.4	50.9 ± 11.5	53.2 ± 12.5
Level of education (n, %)						
Primary (grade 1–6) or lower	499 (33.4)**	551 (39.3)	944 (69.1)	974 (69.7)	903 (66.1)	3871 (55.1)
Middle (grade 7–9) or higher	996 (66.6)	851 (60.7)	422 (30.9)	423 (30.3)	464 (33.9)	3156 (44.9)
Approximate annual household income (n, %)						
Low (<$800 US)	204 (13.6)**	291 (20.8)	959 (70.2)	1036 (74.2)	903 (66.1)	3393 (48.3)
High (≥$800 US)	1291 (86.4)	1111 (79.2)	407 (29.8)	361 (25.8)	464 (33.9)	3634 (51.7)
Height (cm, mean ± SD)	160.1 ± 8.0	160.4 ± 8.1	160.2 ± 7.7	158.2 ± 8.2	156.4* ± 7.7	159.1 ± 8.1
Weight (kg, mean ± SD)	59.5 ± 10.8	59.4 ± 10.3	54.5** ± 8.5	60.7 ± 10.8	55.2 ± 9.0	57.9 ± 10.2
BMI (kg/m^2^, mean ± SD)	23.2 ± 3.6	23.1 ± 3.5	21.2** ± 2.6	24.2 ± 3.8	22.5 ± 3.1	22.8 ± 3.5
Waist circumference (cm, mean ± SD)	77.6 ± 9.2	83.0 ± 10.4	76.9 ± 8.7	85.4 ± 9.4	79.9 ± 8.3	80.5 ± 9.8
Systolic BP (mm Hg, mean ± SD)	122.3 ± 18.2	123.8 ± 19.9	121.2 ± 16.3	139.2** ± 21.6	129.6 ± 21.51	127.2 ± 20.7
Diastolic BP (mm Hg, mean ± SD)	80.0 ± 11.5	79.8 ± 11.5	78.9 ± 10.3	87.7** ± 12.7	84.0 ± 13.0	82.1 ± 12.3
FBG (mmol/l, mean ± SD)	6.2 ± 1.8	5.5 ± 1.1	5.8 ± 1.6	5.3** ± 1.6	5.4 ± 1.4	5.8 ± 1.7

*BMI* body mass index, *BP* blood pressure, *FBG* fasting blood glucose, *SD* standard deviation

* *P* < 0.05, ** *P* < 0.01

As indicated in Table 2, age-standardized prevalence rates of the eight studied CVD risk factors differed significantly by ethnicity. Dai participants had both the highest overall and sex-specific prevalence rates of obesity, central obesity, and hypertension (*P* < 0.01), whereas Jing Po ethnic minority participants had both highest overall and sex-specific prevalence rates of current smoking, SHS exposure, and current drinking levels (*P* < 0.05). Both sexes of Han participants had the highest prevalence of diabetes and physical inactivity (*P* < 0.01), whereas the lowest prevalence rates of diabetes, hypertension, physical inactivity, obesity, and central obesity were found, for both sexes, in the Li Shu population (*P* < 0.01). In all five studied ethnicities, more males than females smoked and consumed alcohol (*P* < 0.01).

**Table 2:** Distribution of weighted age-adjusted prevalence of eight CVD risk factors by ethnicity and sex in rural southwest China

Ethnicity	Hypertension % (95% CI)	Diabetes % (95% CI)	Current smokers % (95% CI)	SHS exposure % (95% CI)	Current drinkers % (95% CI)	Obesity % (95% CI)	Central Obesity % (95% CI)	Physical inactivity % (95% CI)
Han ethnic majority								
Male	26.4 (23.1–29.9)	18.8** (15.5, 21.3)	59.7 (55.6, 62.8)	27.2 (23.3, 33.4)	43.8 (39.8, 47.1)	11.1 (8.6, 12.9)	28.0 (24.6, 31.1)	38.5* (35.5, 41.1)
Female	35.8 (31.9, 39.0)	17.0 **(14.1, 19.5)	1.0 (0.6, 2.1)	42.8 (39.0, 45.9)	2.5 (1.4, 3.8)	8.3 (6.2, 10.4)	33.9 (30.3, 37.2)	48.2** (45.2, 51.0)
All	31.1 (28.3, 33.1)	17.8** (15.5, 19.4)	30.6 (28.4, 32.9)	39.1 (35.2, 41.4)	23.4 (21.1, 25.3)	9.4 (7.8, 10.9)	30.8 (28.3, 33.1)	43.3 **(41.2, 45.2)
Na Xi ethnic minority								
Male	24.0 (21.2, 28.1)	4.6 (2.9, 6.2)	65.8 (62.6, 69.9)	23.0 (19.5, 30.7)	48.1 (44.7, 52.5)	6.7 (5.0, 8.9)	19.6 (17.0, 23.0)	32.4 (29.0, 36.2)
Female	27.8 (25.3, 31.9)	4.3 (2.9, 5.9)	0.7 (0.5, 1.9)	39.2 (35.5, 41.8)	4.5 (2.9, 5.8)	10.5 (8.7, 13.0)	56.4 (52.7, 59.7)	32.3 (28.8, 35.6)
All	26.7 (24.4, 29.0)	4.3 (3.3, 5.4)	30.3 (28.6, 33.4)	31.7 (28.7, 34.6)	24.5 (22.3, 27.1)	8.8 (7.5, 10.5)	39.5 (37.0, 42.1)	32.2 (29.8, 34.7)
Li Shu ethnic minority								
Male	14.1 (12.0, 17.3)	2.0 (1.3, 3.5)	58.8 (54.9, 62.2)	24.5 (19.5, 29.7)	55.7 (51.3, 58.9)	1.8 (1.1, 3.3)	4.4 (3.1, 6.3)	26.0 (23.1, 29.7)
Female	17.3 (15.1, 20.8)	2.1 (1.4, 3.8)	11.0 (8.9, 13.6)	42.0 (37.8, 45.6)	11.6 (9.9, 14.9)	2.1 (1.2, 3.4)	27.4** (24.0, 30.8)	27.1 (24.3, 32.9)
All	16.0 (14.6, 18.5)	2.1 (1.7, 3.3)	34.2 (32.2, 37.2)	36.5 (33.2, 39.6)	33.1 (30.9, 35.9)	2.0 (1.4, 2.9)	16.1 (14.1, 18.0)	26.8 (24.6, 29.1)
Dai ethnic minority								
Male	58.2 **(54.9, 62.1)	7.5 (5.7, 9.6)	66.1 (63.0, 70.1)	13.8 (10.0, 19.1)	63.0 (59.8, 67.1)	14.7** (11.8, 17.2)	52.9** (48.8, 56.3)	36.9 (33.1, 40.7)
Female	50.8** (47.8, 55.0)	8.8 (6.9, 11.1)	0.8 (0.3, 1.6)	32.2 (29.1, 36.0)	2.9 (1.8, 4.3)	19.9 *(16.9, 22.8)	71.7** (67.8, 74.6)	44.0 (40.1, 47.5)
All	54.9** (52.1, 57.3)	8.1 (6.8, 9.6)	33.3 (30.7, 35.7)	27.7 (25.1, 30.9)	32.4 (30.2, 35.2)	17.2** (15.3, 19.0)	62.2** (59.3, 64.5)	41.1 (37.8, 43.2)
Jing Po ethnic minority								
Male	34.4 (30.5, 37.8)	4.2 (2.9, 6.0)	73.0** (68.6, 75.8)	27.9* (24.0, 34.2)	65.2** (61.4, 68.6)	3.9 (2.7, 5.8)	23.1 (20.5, 26.8)	28.8 (25.7, 32.5)
Female	38.6 (35.8, 43.1)	4.5 (2.9, 5.9)	24.0** (20.2, 26.6)	47.1* (41.1, 50.8)	20.5** (17.4, 23.4)	7.5*(5.6, 9.6)	53.7 (50.0, 57.3)	34.5 (30.9, 38.1)
All	36.0 (34.3, 39.5)	4.3 (3.2, 5.3)	48.1** (44.9, 51.0)	42.8* (36.2, 46.2)	42.6** (39.7, 44.8)	5.7 (4.6, 7.1)	38.8 (36.3, 41.2)	31.8 (29.2, 34.2)

*SHS* secondhand smoke

* *P* < 0.05, ** *P* < 0.01

Table 3 shows weighted, age-adjusted prevalence of clustering of CVD risk factors by ethnicity and SES among the study population. Overall, 11.1% of the five ethnicities studied had none of the investigated CVD risk factors, while 66.2% of total participants had clustering of ≥2 risk factors. Clustering of ≥1, ≥2, and ≥ 3 CVD risk factors was found in 92.8, 68.6, and 36.9% of Han, 88.3, 60.2 and 22.6% of Na Xi, 78.8, 50.7 and 18.6% of Li Shu, 96.4, 82.2 and 55.2% of Dai, and 93.8, 73.0 and 37.7% of Jing Po ethnicities, respectively. Dai participants had the highest prevalence of clustering of CVD risk factors (*P* < 0.01). Prevalence of clustering of CVD risk factors increased with age (*P* < 0.01). A clustering of ≥2 and ≥ 3 CVD risk factors was more likely among male participants, participants with low annual household income, and participants with low levels of education (*P* < 0.01).

**Table 3:** Weighted age-adjusted prevalence of clustering of CVD risk factors by ethnicity and socioeconomic status in rural southwest China

Variables	0 (*n* = 840) % (95% CI)	≥1 (*n* = 6187) % (95% CI)	≥2 (*n* = 4456) % (95% CI)	≥3 (*n* = 2273) % (95% CI)
Ethnicity				
Han ethnic majority	7.2 (6.1, 8.4)	92.8 (91.9, 94.0)	68.6 (66.9, 70.6)	36.9 (35.3, 39.1)
Na Xi ethnic minority	11.7 (10.3, 13.5)	88.3 (85.8, 89.4)	60.2 (57.4, 62.5)	22.6 (20.2, 24.7)
Li Shu ethnic minority	21.2 (18.9, 23.8)	78.8 (76.8, 81.1)	50.7 (48.2, 53.3)	18.6 (16.9, 21.0)
Dai ethnic minority	3.6** (2.9, 5.0)	96.4** (95.0, 97.2)	82.2** (79.9, 84.1)	55.2 **(52.4, 58.0)
Jing Po ethnic minority	6.2 (5.0, 7.5)	93.8 (92.5, 95.0)	73.0 (71.0, 75.5)	37.7 (35.2, 40.3)
Sex				
Male	6.4 (5.5, 7.3)	93.6 (92.7, 94.6)	75.5 (74.0, 77.1)	40.3 (38.5, 42.3)
Female	15.1** (13.7, 16.5)	84.9** (83.6, 86.1)	57.7** (56.0, 59.6)	29.9** (28.4, 31.7)
Age				
35–44 years	16.2** (14.3, 17.9)	83.8** (82.1, 85.6)	56.1** (53.9, 58.5)	23.8** (21.6, 25.8)
45–59 years	9.5 (8.4, 11.0)	90.5 (89.1, 91.6)	67.8 (65.6, 69.6)	35.9 (34.1, 38.0)
≥ 60 years	6.2 (5.2, 7.6)	93.8 (92.4, 94.8)	77.5 (75.2, 79.4)	46.6 (44.2, 49.3)
Level of education				
Primary (grade 1–6) or lower	9.9 (9.0, 11.1)	90.1 (88.7, 91.1)	70.2* (66.2, 71.7)	37.2** (35.2, 38.8)
Middle (grade 7–9) or higher	11.6 (10.2, 13.1)	88.4 (87.3, 89.8)	63.0 (61.0, 66.2)	32.1 (30.3, 34.6)
Approximate annual household income (%)				
Low (<$800 US)	9.7 (9.0, 11.2)	90.3 (88.7, 91.4)	69.2** (67.0, 71.3)	36.9* (34.7, 38.2)
High (≥$800 US)	12.2 (9.9, 13.2)	88.8 (87.4, 90.1)	63.8 (62.0, 66.1)	33.2 (31.6, 35.3)
All	11.1 (9.8, 12.4)	88.9 (87.4, 90.2)	66.2 (65.4, 67.8)	34.8 (33.8, 36.2)

* *P* < 0.05, ** *P* < 0.01

## Discussion

The findings show prevalence of CVD risk factors in rural southwest China varied by ethnicity, and clustering of CVD risk factors was independently associated with ethnicity and individual SES.

The top two CVD risk factors in the study population were prevalence of current smoking and current drinking. Further, across all five studied ethnicities, females had markedly lower prevalence of smoking and drinking behaviors than males. This finding aligns with other studies previously conducted in Asia (including China) as well as prior research in western nations [16, 26, 27]. Additionally, in the present study ethnic minority participants smoked and drank more than Han majority participants. This may in significant part result from differing cultural practices and customs that possibly in turn influence smoking and drinking behaviors. Such ethnic variations were similarly recorded in studies conducted in western countries [12, 28]. However, the prevalence of current smoking and drinking in the present study was significantly higher than previously reported rates in both other parts of China [7] as well as other Asian countries [29]. The findings thus reveal smoking and drinking are highly prevalent, especially among men belonging to ethnic minority groups in rural southwest China.

Previous research has established that SHS exposure is a contributing cause of CVD [30]. Females were at a higher risk of exposure to SHS than males in the present study. Among the five studied ethnicities, Jing Po ethnic minority female participants had both the highest overall and sex-specific prevalence of current smoking and SHS exposure. This may in part be attributed to geography: nearly all Jing Po Yunnan residents live in remote mountainous regions and have accordantly less access to education and health information on the hazards of tobacco use. Further, the prevalence of exposure to SHS in Jing Po females (45.6%) was greater than the prevalence rates observed in other studies [31], indicating exposure to SHS is an especially serious health challenge for Jing Po women. The results thereby suggest it is critical to strengthen women’s awareness of the hazards of tobacco. Moreover, they suggest that targeted and comprehensive smoke-free laws and legislation should be enacted in rural China to prevent women from exposure to SHS.

Relative to the other four ethnic groups in the present study, Han participants had increased risk for diabetes. This may in part result from the fact that Han participants also had the highest annual household income in the studied regions: several Chinese and Asian studies have demonstrated that high income is associated with high prevalence of diabetes [32, 33]. Furthermore, Han participants had lower physical activity levels than the other four studied ethnic groups. Varying lifestyle behaviors among Chinese ethnic groups may lie behind this activity level difference. Major improvements in living conditions in rural China have previously been associated with large lifestyle changes, including a rise in sedentary behavior [10]. However, the precise causes of the considerable ethnic differences in diabetes and physical activity levels observed in the present study necessitate further research.

The present study revealed significant ethnic differences in prevalence of hypertension, with Dai ethnic minority participants with the highest prevalence of hypertension. Such ethnic variation was also uncovered previously in China [15]. However, Dai participants in the present survey had a significantly higher prevalence of hypertension than that observed in previous Chinese surveys [7, 8]. This may result from the fact that Dai participants had the highest prevalence of both obesity and central obesity, and obesity and central obesity are central causes of hypertension [3, 10]. The ethnic differences in prevalence of hypertension uncovered in the present study indicate ethnicity is a central determinant for hypertension, and further research is needed.

Overall, 75.5% of male participants and 57.7% of female participants in the present study clustered two or more investigated CVD risk factors, a rate higher than that found in previous Chinese studies conducted in Nanjing City (45.2 and 24.6%) [7] and Jilin City (27.9 and 19.8%) [34], as well as higher than studies of ethnic minorities in the Xinjiang Uygur autonomous region (29.5 and 48.8%) [15], indicating clustering of CVD risk factors was comparatively common in rural southwest China. Dai participants had the highest level of CVD risk factor clusters in the present study, suggesting they are at the highest risk of developing CVD relative to the other four studied ethnicities. However, the majority of previous research conducted in China did not consider SHS exposure and physical activity level, so the full range of risk factors attributable to CVD was not captured in these prior studies. Thus, the clustering of CVD risk factors in those studies was likely underestimated.

Men were more likely to have two or more CVD risk factors clustered in the present study, and clustering of CVD risk factors increased with age. This result is in line with previous studies in China [7–9, 14]. Moreover, in all five ethnic minorities studied, a low annual household income and low education level were associated with a high risk of CVD risk factors clustered. The socioeconomic differential measured by income and educational level accords with previous studies across the globe, in both developed and developing countries [12, 28, 34]. The findings suggest comprehensive lifestyle interventions should be initiated at a young age, and future CVD prevention and intervention strategies should focus in particular on those with low household income and education levels.

The findings of the present study are limited in two ways. First, the present study on the clustering of CVD risk factors was limited by its focus on eight selected risk factors. Further research is needed to examine the contribution of other risk factors to CVD in rural China. Namely, the study lacked diet-related data, and diet may be an important factor influencing CVD. Second, the study gathered and analyzed cross-sectional data, so causal relationships cannot be determined.

## Conclusion

The findings indicate a high prevalence of each studied CVD risk factor as well as high clustering of CVD risk factors in rural southwest China. As ethnicity and individual SES were found to have significant impacts on prevalence of CVD risk factors and their clustering, culturally tailored interventions are needed to improve CVD prevention and control strategies in rural southwest China.

## Data Availability

The datasets used and/or analyzed during the current study is available from the corresponding author on reasonable request.

## Abbreviations

BMI: Body mass index
BP: Blood pressure
CI: Confidence interval
CVD: Cardiovascular disease
DBP: Diastolic blood pressure
FBG: Fasting blood glucose
SBP: Systolic blood pressure
SD: Standard deviation
SES: Socioeconomic status
SHS: Secondhand smoke
WC: Waist circumference
WHO: World Health Organization

## References

[1] GBD 2016 Causes of Death Collaborators (2017). Global, regional, and national age-sex specific mortality for 264 causes of death, 1980–2016: a systematic analysis for the Global Burden of Disease Study 2016. Lancet.

[2] GBD 2013 Mortality and Causes of Death Collaborators (2015). Global, regional, and national age–sex specific all-cause and cause-specific mortality for 240 causes of death, 1990–2013: a systematic analysis for the Global Burden of Disease Study 2013. Lancet.

[3] Stevens W, Peneva D, Li JZ, Liu LZ, Liu G, Gao R, Lakdawalla DN (2016). Estimating the future burden of cardiovascular disease and the value of lipid and blood pressure control therapies in China. BMC Health Serv Res.

[4] Moran A, Gu D, Zhao D, Coxson P, Wang YC, Chen CS (2010). Future cardiovascular disease in China: markov model and risk factor scenario projections from the coronary heart disease policy model-China. Circ Cardiovasc Qual Outcomes.

[5] GBD 2016 Risk Factors Collaborators (2017). Global, regional, and national comparative risk assessment of 84 behavioural, environmental and occupational, and metabolic risks or clusters of risks, 1990–2016: a systematic analysis for the Global Burden of Disease Study 2016. Lancet.

[6] GBD 2015 Tobacco Collaborators (2017). Smoking prevalence and attributable disease burden in 195 countries and territories, 1990–2015: a systematic analysis from the Global Burden of Disease Study 2015. Lancet.

[7] Hong X, Ye Q, He J, Wang Z, Yang H, Qi S, Chen X, Wang C, Zhou H, Li C, Qin Z, Xu F (2018). Prevalence and clustering of cardiovascular risk factors: a cross-sectional survey among Nanjing adults in China. BMJ Open.

[8] Wu J, Cheng X, Qiu L, Xu T, Zhu G, Han J, Xia L, Qin X, Cheng Q, Liu Q (2016). Prevalence and Clustering of Major Cardiovascular Risk Factors in China: A Recent Cross-Sectional Survey. Medicine (Baltimore).

[9] The Collaborative Study Group on Trends of Cardiovascular Disease in China and Preventive Strategy (2001). Current status of major cardiovascular risk factors in Chinese populations and their trends in the past two decades. Chin J Cardiol.

[10] Wu Y, Benjamin EJ, MacMahon S (2016). Prevention and Control of Cardiovascular Disease in the Rapidly Changing Economy of China. Circulation.

[11] Li Y, Wang DD, Ley SH, Howard AG, He Y, Lu Y, Danaei G, Hu FB (2016). Potential Impact of Time Trend of Life-Style Factors on Cardiovascular Disease Burden in China. J Am Coll Cardiol.

[12] Boykin S, Diez-Roux AV, Carnethon M, Shrager S, Ni H, Whitt-Glover M (2011). Racial/ethnic Heterogeneity in the Socioeconomic Patterning of CVD Risk Factors: in the United States: The Multi-Ethnic Study of Atherosclerosis. J Health Care Poor Underserved.

[13] Perini W, Snijder MB, Peters RJG, Kunst AE (2018). Ethnic disparities in estimated cardiovascular disease risk in Amsterdam, the Netherlands: The HELIUS study. Neth Hear J.

[14] Tao J, Ma Y-t, Xiang Y, Xie X, Yang Y-n, Li X-m, Fu Z-Y, Ma X, Liu F, Chen B-d, Yu Z-x, Chen Y (2013). Prevalence of major cardiovascular risk factors and adverse risk profiles among three ethnic groups in the Xinjiang Uygur Autonomous Region, China. Lipids Health Dis.

[15] Li N, Wang H, Yan Z, Yao X, Hong J, Zhou L (2012). Ethnic disparities in the clustering of risk factors for cardiovascular disease among the Kazakh, Uygur, Mongolian and Han populations of Xinjiang: a cross-sectional study. BMC Public Health.

[16] Le C, Xinan W, Goyal A, Han Y, Cui W, Xiao X, He J, Zhao K, Song Y, Jiao F (2012). Patterns and socioeconomic influences of tobacco exposure in tobacco cultivating rural areas of Yunnan Province, China. BMC Public Health.

[17] Cai L, Dong J, Cui WL, You DY, Golden AR (2017). Socioeconomic differences in prevalence, awareness, control and self-management of hypertension among four minority ethnic groups, Na xi, Li Shu, Dai and Jing Po, in rural Southwest China. J Hum Hypertens.

[18] Perloff D, Grim C, Flack J, Frohlich ED, Hill M, McDonald M, Morgenstern BZ (1993). Human blood pressure determination by sphygmomanometry. Circulation.

[19] WHO (2005). WHO STEPS surveillance manual: the WHO STEPwise approach to chronic disease risk factor surveillance / noncommunicable diseases and mental health.

[20] Le C, Lin L, Jun D, Jianhui H, Keying Z, Wenlong C, Ying S, Tao W (2013). The economic burden of type 2 diabetes mellitus in rural southwest China. Int J Cardiol.

[21] Chobanian AV, Bakris GL, Black HR, Cushman WC, Green LA, Izzo JL, Jones DW, Materson BJ, Oparil S, Wright JT, Roccella EJ (2003). National Heart, Lung, and Blood Institute Joint National Committee on Prevention, Detection, Evaluation, and Treatment of High Blood Pressure; National High Blood Pressure Education Program Coordinating Committee. The seventh report of the joint National Committee onPrevention, detection, evaluation, and treatment of high blood pressure: the JNC 7 report. JAMA.

[22] WHO. Definition, Diagnosis and classification of diabetes mellitus and its complications, part 1: diagnosis and classification of diabetes mellitus; World Health Organization: Geneva, 1999.

[23] WHO/LASA/IOTF (2000). The Asia Pacific Perspective: Redefining obesity and its treatment.

[24] World Health Organization (2015). Physical activity and adults: recommended levels of physical activity for adults aged 18–64 years.

[25] Khanal MK, Mansur Ahmed MSA, Moniruzzaman M, Banik PC, Dhungana RR, Bhandari P, Devkota S, Shayami A (2018). Prevalence and clustering of cardiovascular disease risk factors in rural Nepalese population aged 40-80 years. BMC Public Health.

[26] Li L, Borland R, Yong H-H, Fong GT, Bansal-Travers M, Quah ACK, Sirirassamee B, Omar M, Zanna MP, Fotuhi O (2010). Predictors of smoking cessation among adult smokers in Malaysia and Thailand: Findings from the International Tobacco Control Southeast Asia Survey. Nicotine Tob Res.

[27] Caetano R, Vaeth PA, Canino G (2016). Prevalence and predictors of drinking, binge drinking, and related health and social problems in Puerto Rico. Am J Addict.

[28] Cook WK, Caetano R (2014). Ethnic Drinking Cultures, Gender, and Socioeconomic Status in Asian American and Latino Drinking. Alcohol Clin Exp Res.

[29] Hong JW, Noh JH, Kim D-J (2017). The prevalence of and factors associated with high-risk alcohol consumption in Korean adults: The 2009–2011 Korea National Health and Nutrition Examination Survey. PLoS One.

[30] Dunbar A, Gotsis W, Frishman W (2012). Secondhand tobacco smoke and cardiovascular disease risk: an epidemiological review. Cardiol Rev.

[31] Sung H-Y, Chang L-C, Wen Y-W, sai Y-WT (2014). The costs of smoking and secondhand smoke exposure in Taiwan: a prevalence-based annual cost approach. BMJ Open.

[32] Pubudu De Silva A, Padmal De Silva SH, Liyanage IK, Rajapakse LC, Jayasinghe KS, Katulanda P, Wijeratne CN, Wijeratne S (2012). Social, cultural and economical determinants of diabetes mellitus in Kalutara district, Sri Lanka: a cross sectional descriptive study. Int J Equity Health.

[33] Xu Y, Wang L, He J, Bi Y, Li M, Wang T, Wang L, Jiang Y, Dai M, Lu J, Xu M, Li Y, Hu N, Li J, Mi S, Chen CS, Li G, Mu Y, Zhao J, Kong L, Chen J, Lai S, Wang W, Zhao W (2013). Ning G; 2010 China Noncommunicable Disease Surveillance Group. Prevalence and control of diabetes in Chinese adults. JAMA.

[34] Yu J, Ma Y, Yang S, Pang K, Yu Y, Tao Y, Jin L (2016). Risk Factors for Cardiovascular Disease and Their Clustering among Adults in Jilin (China). Int J Environ Res Public Health.

